# Analysis of Flow Characteristics of the Blood Flowing through an Inclined Tapered Porous Artery with Mild Stenosis under the Influence of an Inclined Magnetic Field

**DOI:** 10.1155/2014/797142

**Published:** 2014-02-25

**Authors:** Neetu Srivastava

**Affiliations:** Department of Mathematics, Amrita Vishwa Vidyapeetham (University), Karnataka 560 035, India

## Abstract

Analytical investigation of MHD blood flow in a porous inclined stenotic artery under the influence of the inclined magnetic field has been done. Blood is considered as an electrically conducting Newtonian fluid. The physics of the problem is described by the usual MHD equations along with appropriate boundary conditions. The flow governing equations are finally transformed to nonhomogeneous second-order ordinary differential equations. This model is consistent with the principles of magnetohydrodynamics. Analytical expressions for the velocity profile, volumetric flow rate, wall shear stress, and pressure gradient have been derived. Blood flow characteristics are computed for a specific set of values of the different parameters involved in the model analysis and are presented graphically. Some of the obtained results show that the flow patterns in converging region (*ξ* < 0), diverging region (*ξ* > 0), and nontapered region (*ξ* = 0) are effectively influenced by the presence of magnetic field and change in inclination of artery as well as magnetic field. There is also a significant effect of permeability on the wall shear stress as well as volumetric flow rate.

## 1. Introduction

Blood is a thick red liquid circulating in the blood vessels. It has a strong nourishing effect on the human body and serves as one of the basic substances constituting the human body. Blood is a suspension of cellular elements—red blood cells (erythrocytes), white blood cells (leukocytes), and platelets in an aqueous electrolyte solution called plasma. Plasma contains 90% of water and 7% of principal proteins (albumin, globulin, lipoprotein, and fibrinogen). The volumetric fraction of the erythrocytes is 45% of the total volume of blood in normal blood, which defines an important variable called hematocrit. Of the remaining blood cells, the leukocytes are less than (1/600)th of the red blood cells and the platelets concentration is (1/20)th of the red blood cells.

Hemodynamics describes the mechanism that affects the dynamics of blood circulation in the human body. Under this, atherosclerosis, a leading cause of death in many countries, is one of the phenomena in which the blood circulation will get affected by the intimal thickening of stenos artery. A blockage by atherosclerosis, which is a progressive vascular disease that causes accumulation of fatty substances, calcium, fibrin, cellular waste, and cholesterol, also known as plaque, inside the walls of the arteries, leads to the narrowing of the walls of the arteries and causes the condition known as carotid artery disease.

Severe stenosis may cause critical flow conditions of blood by reducing the blood supply and resulting in serious consequences called carotid artery blockage which is one of the major contributing factors to strokes. This is because when the plaque hardens and narrows down the arteries completely, the blood supply and oxygen to the brain are reduced. Without proper blood or oxygen supply, the cells in the brain start to die. This leads to the loss of brain function and permanent brain damage or the death of an individual. However, the chances of developing carotid artery disease may increase as a result of carotid aneurysm disease, fibromuscular dysplasia, or diabetes. In certain cases, plaque built up in the arteries can break off, travel through the bloodstream, and get lodged in some blood vessels in the brain. This can trigger off a transient ischemic attack. It is thus vital to watch out for the symptoms of a carotid artery blockage, so that necessary steps can be taken before the individual's condition worsens.

The problem of fluid mechanics has attracted the attention of many investigators. If fluid is considered as blood, then the equation of motion suggested by the [1, 2] can be modified and can be used to gather an accurate knowledge of the mechanical properties of the vascular wall together with the flow characteristics of blood, in order to have a full understanding of the development of these diseases, which will assist bioengineers who are engaged in the design and construction of improved artificial organs and also helps in the treatment of vascular diseases. Perhaps the actual cause of abnormal growth in arteries is not completely clear to the theoretical investigators but its effect on the cardiovascular system has been determined by studying the flow characteristics of blood in the stenosed area. Although the applicability of a purely mechanical model for such a physiological problem has obvious limitations, vascular rheology together with hemodynamic factors is predominant in the development and progression of arterial stenosis.

The idea of electromagnetic fields in medical research was firstly given by Kolin [3], and later Korchevskii and Marochnik [4] discussed the possibility of regulating the movement of blood in human system by applying magnetic field. Vardanyan [5] studied the effect of magnetic field on blood flow theoretically and his work was later corroborated by Sud et al. [6] and Suri and Suri Pushpa [7] by considering different models. It was observed by these authors that the effect of magnetic field is to slow down the speed of blood. However, the published literature lacks the analysis of magnetic effect on blood flow through an inclined branched artery. If a magnetic field is applied to a moving electrically conducting liquid, it induces electric and magnetic fields. The interaction of these fields produces a body force known as Lorentz force which has a tendency to oppose the movement of the liquid. For the flow of blood in arteries with arterial disease like arterial stenosis or arteriosclerosis, the influence of magnetic field may be utilized as a blood pump in carrying out cardiac operations, and in addition to this, the effects of vessels tapering together with the shape of stenosis on the flow characteristics seem to be equally important and deserve special attention.

Chakraborty et al. [8] discussed the suspension model blood flow through an inclined tube with an axially nonsymmetric stenosis. Eldesoky [9] studied the slip effect of unsteady magnetic field on pulsatile blood flow through porous medium in an artery under the effect of body acceleration. Bali and Awasthi [10] studied the Newtonian blood flow through the tapered arteries. The important contributions of recent years to the topic are referenced in the literature [11–19]. However, the published work still lacks the analysis of the blood flow characteristics through an inclined artery with the applied magnetic field. This problem has taken a care for this.

In this paper, we have considered the effects of arterial wall parameters on the flow of Newtonian fluid (as a blood model) through an inclined tapered porous artery with axially nonsymmetric mild stenosis under effect of an external inclined magnetic field (Figure 2).

## 2. Formulation of the Problem

**Figure 1:**
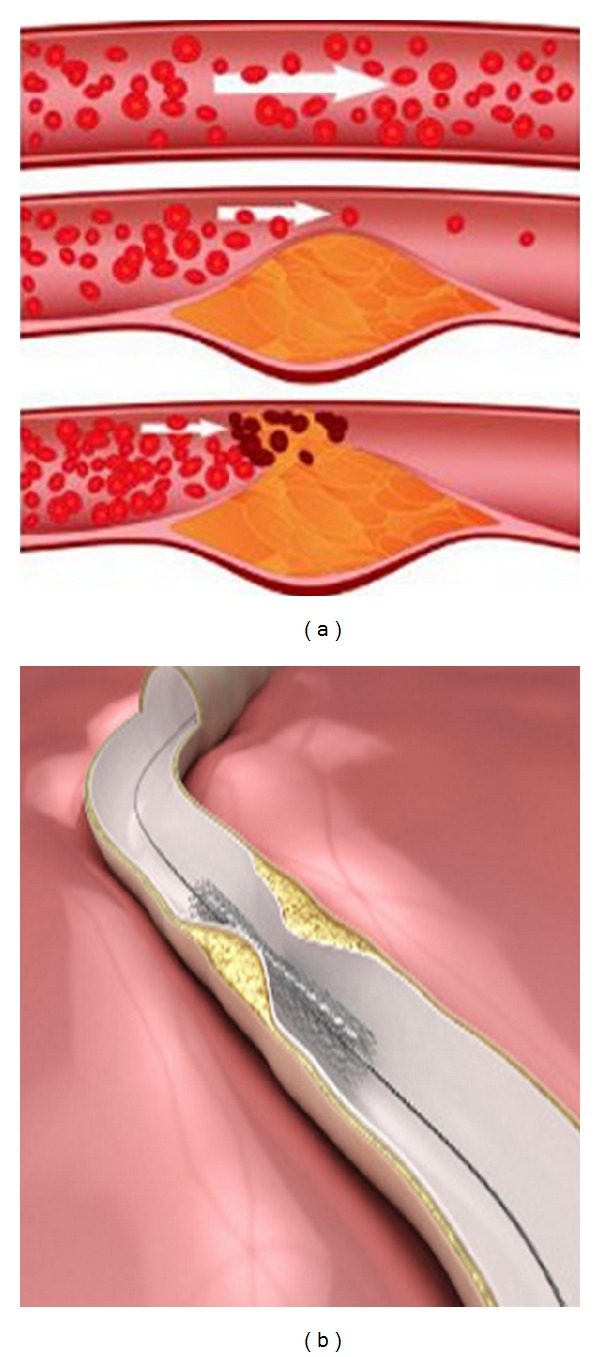
Atherosclerosis.

**Figure 2:**
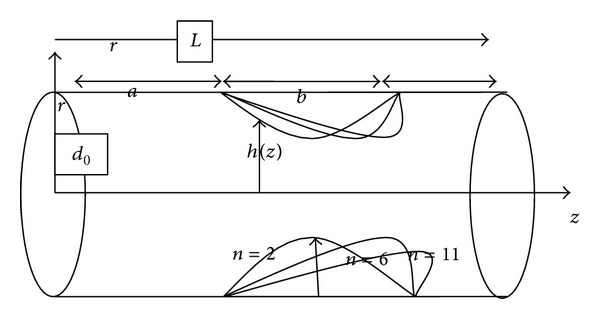
Schematic diagram for the porous artery with the different stenosis.

**Figure 3:**
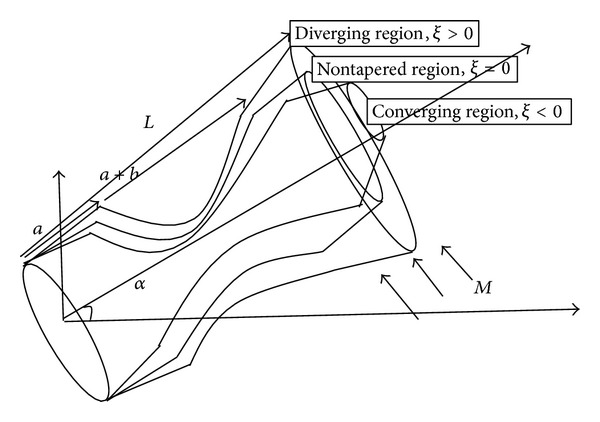
Schematic diagram for the tapered porous artery inclined at an angle *α* and with the applied magnetic field inclined at *θ*.

Consider an axisymmetric steady flow of blood with a constant viscosity *μ* and density *ρ* in a cylindrical porous artery of radius *d*_0_ and length *L* inclined at an angle *α* represented in Figure 1. Let *θ* be an angle of inclined magnetic field externally applied to an inclined porous artery (Figure 3). Let (*r*, *ϕ*, *z*) be the cylindrical polar coordinates with *r* = 0 as the axis of symmetry of the tube. Let us define *V*_*r*_,  *V*_*ϕ*_,  *V*_*z*_ as components of velocity. Since the flow is axisymmetric, so variation in the blood flow characteristic is independent of an azimuthal angle. The geometry of stenosis is defined by

(1) $$\begin{array}{c} h\left(z\right)=\{\begin{array}{cc} d\left(z\right)[1-\eta (b^{n-1}\left(z-a\right) & \\ \;\;\;\;\;\;\;-{\left(z-a\right)}^{2})], & a\leq z\leq a+b,\\ d\left(z\right), & “\text{otherwise}”, \end{array} \end{array}$$

with

(2) $$\begin{array}{c} d\left(z\right)=d_{0}+\xi z, \end{array}$$

where *d*(*z*) is the radius of tapered arterial segment in the stenotic region, *d*_0_ is the radius of the nontapered artery in the nonstenotic region, *ξ* is the tapering parameter, *b* is the length of the stenosis, and *n*( ≥ 2) is a parameter determining the shape constriction profile and referred to as a shape parameter. The parameter *η* is defined by

(3) $$\begin{array}{c} \eta =\frac{\delta n^{\left(1/\left(n-1\right)\right)}}{d_{0}b^{n}\left(n-1\right)}, \end{array}$$

where *δ* denotes the maximum height of the stenosis located at

(4) $$\begin{array}{c} z=a+\frac{b}{n^{\left(1/\left(n-1\right)\right)}}. \end{array}$$

The equation describing the steady flow of blood through the cylindrical artery inclined at an angle *α* can be written as follows:

(5) $$\begin{array}{c} \nabla \cdot \overset{-}{V}=0, \end{array}$$

(6) $$\begin{array}{c} \rho \overset{-}{F}=-\nabla p+\mu \nabla^{2}\overset{-}{V}-\frac{\mu \overset{-}{V}}{\kappa }+\overset{-}{J}X\overset{-}{B}. \end{array}$$

Boundary conditions for the given problem can be written as follows:

(7) $$\begin{array}{c} u=0\;\text{at}\;r=h\left(z\right),\\ \frac{\partial u}{\partial r}=0\;\text{at}\;r=0,\\ \;u\;\text{is}\;\text{finite}\;\text{at}\;r=0. \end{array}$$

Based on the assumption made for the flow, (5) can be reduced to

(8) $$\begin{array}{c} \rho g\sin\alpha =-\frac{\partial p}{\partial z}+\mu \left(\frac{\partial^{2}u}{{\partial r}^{2}}+\frac{1\partial u}{r\partial r}\right)-\frac{\mu u}{\kappa }-\sigma B_{0}^{2}u, \end{array}$$

where *α* is the angle of inclination of an artery, *ρ* is the fluid density, *B*_0_ is an applied magnetic field with an inclination *θ*, *κ* is the permeability of the porous medium, *g* is the acceleration due to gravity, and *μ* is the viscosity of the blood.

## 3. Equation of Motion

Introducing the nondimensional variables as follows:

(9) z′=zd,  r′=rd0,    h′=hd0,  v′=bvδu0,  u′=uu0,      h′=hd0,  p′=pd02bμu0,Re=ρu0d0μ,  κ′=κd02,Fr=u02gd0,  M=σd02B02μ,

where *Re*, Fr, and *M* define the Reynolds number, Froude number, and Hartmann number, respectively. Substituting (9) in (8), we can have a dimensionless form for (8) as follows:

(10) ReFrsinα=−∂p∂z+μ(∂2u∂r2+1∂ur∂r)−(1κ+M2cos⁡2θ)u.

As the flow is steady and axisymmetric, let the solution for *u*(*r*, *t*) and *p* be set in the forms:

(11) $$\begin{array}{c} u\left(r,t\right)=\overset{-}{u}\left(r\right),\;\;-\frac{\partial p}{\partial z}=P, \end{array}$$

where *P* is a constant. Substituting (11) in (10), we can have an ordinary differential equation as follows:

(12) d2udr2+1durdr−β2u=ReFrsinα−P,

where *β* ^2^ = (1/*κ* + *M* ^2^cos⁡^2^ *θ*).

The solution for the equation of motion (12) can be written as follows:

(13) u(r)=(κκ+M2cos⁡2θ)(ReFrsinα−P)[I0(βr)I0(βh)−1],

where *I*_0_ is the modified Bessel function of the zero order. The volumetric flow rate can be given by

(14) $$\begin{array}{c} Q=2\pi \int_{0}^{h}ru\;dr. \end{array}$$

Substituting (13) with (14) and integrating it, we get

(15) Q=1β2(ReFrsinα−P)(−h22+hβI1(βh)I0(βh)),

where *I*_1_ is the Bessel function of the order one. The expression for the wall shear stress at *r* = *h* can be written as follows:

(16) τr=μβ(ReFrsinα−P)(I1(βh)I0(βh)).

## 4. Results

In a converging and diverging region, for the analysis of the salient features of the blood flow, the effect of vital parameters defining flow geometries and fluid behaviour such as permeability, Hartmann number, and inclination angle of artery (*α*) as well as the inclination of magnetic field (*θ*) on the flow characteristic such as wall shear stress, shear stress at stenosis throat, axial velocity, and the volumetric flow rate are discussed numerically with computational illustrations. All graphs are plotted for the values of *σ* = *b*/*a* = 0, *b* = 1, *ξ* = tan*ϕ* = 0.002, 0,  −0.002, *Re* = 0.1, Fr = 0.1, *M* = 2, 3, and 4, shape parameter *n* = 2,  6, and 11, and stenosis height = 0 to .20 using Mathematica software.

**Figure 4:**
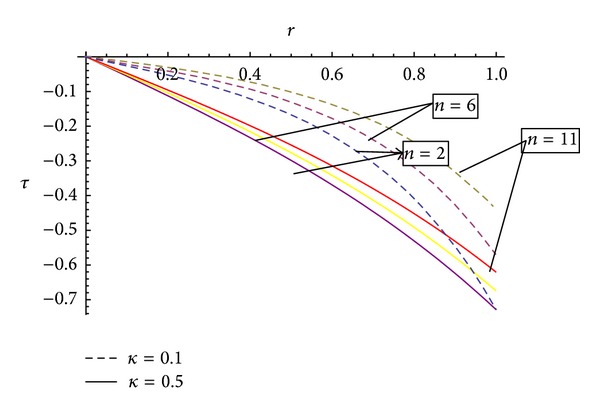
Effect of permeability on the shearing stress for different values of shape parameter *n* and permeability.

Figure 4 indicates that the behaviour of wall shear stress is inversely proportional to the permeability, and with the increase of shape parameter, the wall shear stress increases. For the symmetric stenosis (*n* = 2), the wall shear stress is less as compared to the asymmetric stenosis (*n* = 6, 11). Also, with the decrease of permeability parameter, the graph tends towards the parabolic nature, and also shear stress decreases with an increase of permeability parameter.

**Figure 5:**
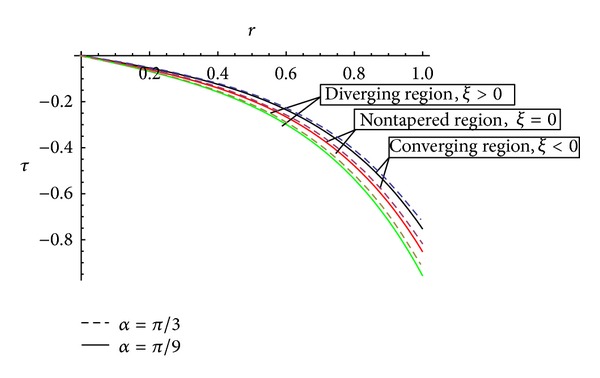
Effect of artery inclination on the shearing stress for different values of tapering angle  *ξ*.

Figure 5 represents the behaviour of shearing stress with the inclination of artery for the different values of tapering angle. It has been observed that for the converging region (*ξ* < 0), the stress will be more as compared to the diverging region (*ξ* > 0) and the nontapered region (*ξ* = 0) that is. Wall shear stress increases with an increase in tapering angle (*ϕ*) which is in agreement with Chaturani and Prahlad's model [20].

**Figure 6:**
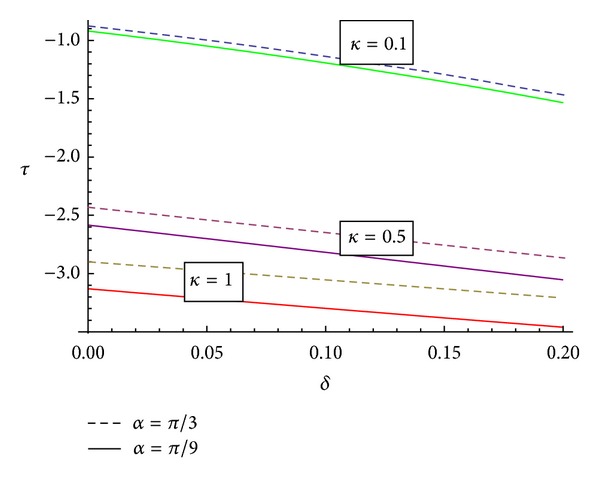
Variation of shearing stress at the stenosis throat *δ* with different inclination for “*α*” different values of permeability parameter “*κ*”.

Figure 6 represents the behaviour of shearing stress with the stenosis throat for the different values of inclination of artery. It has been observed that with the increase of “*α*”, the shearing stress “*τ*” increases and also the variation of permeability parameter affects the stress inversely. This graph also reveals that with the increase of stenosis height the shearing stress increases. It rapidly decrease in the downstream and maximum value is near the end of the stenosis. This is in agreement with Young's model [19].

**Figure 7:**
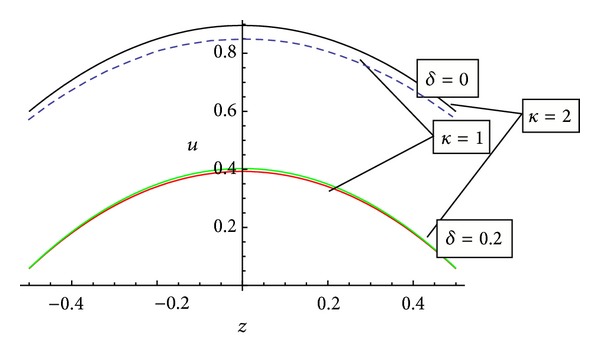
Variation of axial velocity *u* with *z* and height of stenosis *δ* for different values of permeability.

Figure 7 represents the behaviour of axial velocity with the stenosis height for different values of permeability. It has been observed that the axial velocity possesses reverse behaviour on the either side of the centreline of the artery. This figure also shows an increase in axial velocity with the increase of permeability.

**Figure 8:**
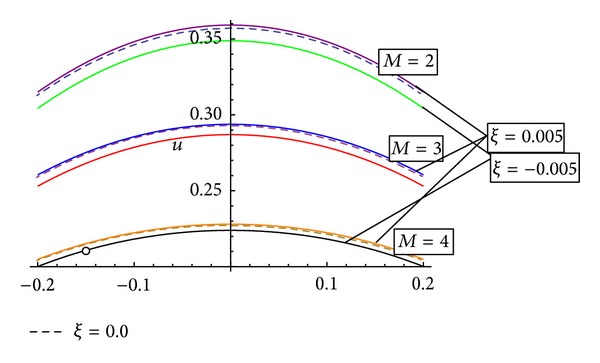
Variation of axial velocity *u* with magnetic field for different values of tapering angle.

Figure 8 represents the behaviour of axial velocity with the magnetic field for a different tapering angle. It has been observed that with the increase of magnetic field the axial velocity shows a reverse behaviour. It is observed that with the increase of magnetic field, the curves representing the axial flow velocity do shift towards the origin for a converging region, while they shift away from the origin for a nontapered and diverging tapered artery.

**Figure 9:**
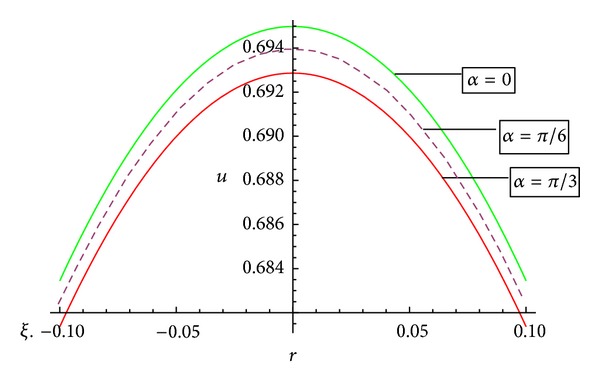
Variation of axial velocity *u* with *z* for different values of artery angle *α*.

Figure 9 represents the behaviour of axial velocity for different values of artery inclination and shows the remarkable changes with the inclination of artery. It has been observed that with the increase of inclination of artery, the curve representing the axial flow velocities does shift towards the origin.

**Figure 10:**
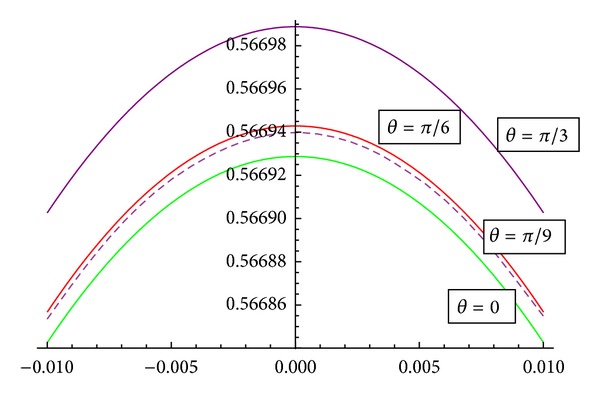
Axial velocity with the inclination of magnetic field *θ* for *Re* = 0.1, Fr = 0.1, *κ* = 2, *M* = 2, and *α* = *π*/3.

Figure 10 represents that the axial velocity variation with the inclination of magnetic field.It has been observed that with the increase of inclination of magnetic field, the curve does shift towards the origin.

**Figure 11:**
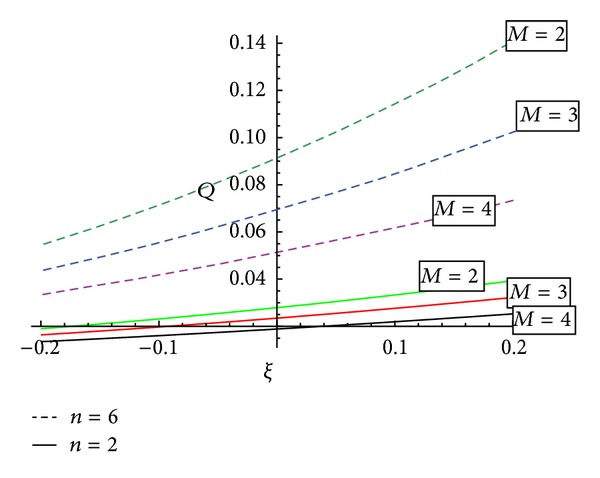
Variation of volumetric flow rate with the tapering angle, for different values of *M* and for fixed values of *Re* = 0.1, Fr = 0.1, *α* = *π*/3, and *κ* = 2.

Figure 11 reveals that the variation of volumetric flow rate for an inclined artery will decrease with the Hartmann number for the fixed values of Reynolds number *Re* = 0.1, Froude number Fr = 0.1, artery inclination = *π*/3, and permeability parameter *κ* = 2, but it will be greater in diverging region as compared to converging region.

**Figure 12:**
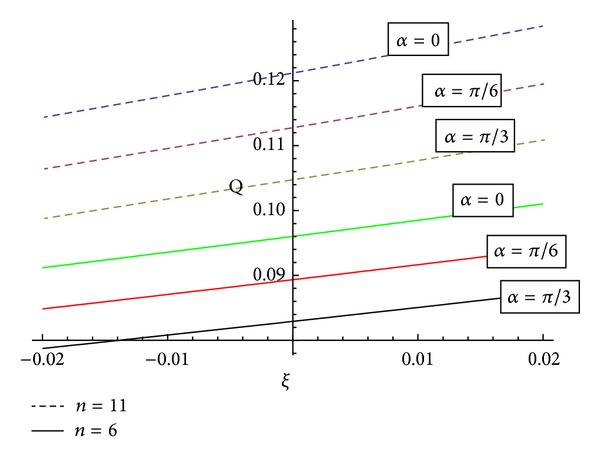
Variation of volumetric flow rate with the angle of inclination *α* of artery for converging, diverging region, and nontapered region.

Figure 12 reveals the information about that variation of volumetric flow rate with the angle of inclination “*α*” of artery for converging, diverging region, and nontapered region. It shows that with the increase of “*α*” the volumetric flow rate decreases.

**Figure 13:**
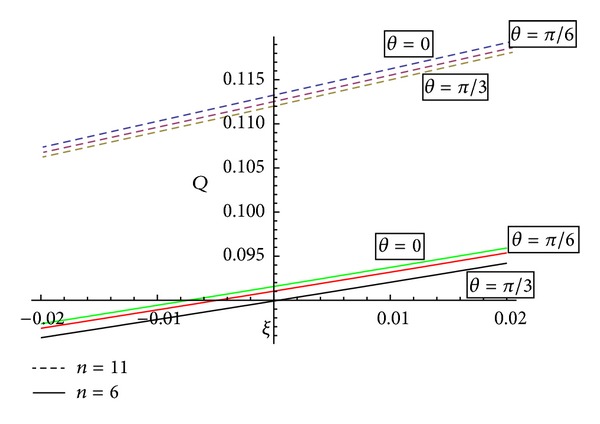
Variation of volumetric flow rate with the angle of magnetic field *θ* for converging, diverging region, and nontapered region.

Figure 13 reveals that the variation of volumetric flow rate with the angle of magnetic field *θ* will increase for all converging (*ξ* < 0), diverging region (*ξ* > 0), and nontapered region (*ξ* = 0).

## 5. Conclusion

The present study deals with the analysis of flow characteristics of the blood flowing through an inclined tapered porous artery with mild stenosis under the influence of an inclined magnetic field. This investigation can play a vital role in the determination of axial velocity, shear stress, and fluid acceleration in particular situations. Since this study has been carried out for a situation when the human body is subjected to an external magnetic field, it bears the promise of significant application in magnetic or electromagnetic therapy, which has gained enough popularity. The study is also useful for evaluating the role of porosity. The analysis is carried out by employing appropriate analytical methods, and some important predictions have been made basing upon the study. The main findings of the present mathematical analysis are as follows.

- The behaviour of wall shear stress is inversely proportional to the permeability, and with the increase of shape parameter, the wall shear stress increases. Wall shear stress increases with an increase of tapering angle (*ϕ*) which is in agreement with Chaturani and Prahlad's model [20].
- Shearing stress “*τ*” rapid decrease in the downstream and maximum value is near the end of the stenosis. This is in agreement with Young's model [19].
- Concerning the behaviour of axial velocity with the stenosis height for different values of permeability, it has been observed that the axial velocity possesses reverse behaviour on either side of the centreline of the artery.
- With the increase of magnetic field, the axial velocity shows a reverse behaviour. It is observed that with the increase of magnetic field, the curves representing the axial flow velocity do shift towards the origin for a converging region, while they shift away from the origin for a nontapered and diverging tapered artery.
- Axial velocity shows the remarkable changes with the inclination of artery. It has been observed that with the increase of inclination of artery, the curve representing the axial flow velocities does shift towards the origin.
- The volumetric flow rate increases with the increase of shape parameter  *n*  and decreases with the increase of Hartmann number. Also, Figure 11 shows an increasing behaviour of volumetric flow rate from converging to diverging region.
- The variation of volumetric flow rate for an inclined artery will be greater in diverging region as compared to converging region.
- Volumetric flow rate varies inversely with the angle of inclination “*α*” of artery for converging, diverging region, and nontapered region.
- With the angle of inclination of magnetic field *θ*, the volumetric flow rate will increase and will be more for diverging region as compared to converging region.
